# The Treatment of the Deciduous Molars

**Published:** 1904-01

**Authors:** C. N. Johnson

**Affiliations:** Chicago, Ill.


### THE TREATMENT OF THE DECIDUOUS MOLARS.

By C. N. Johnson, L.D.S., Chicago, Ill.

Read before the Second District Dental Society of the State of New York.

The chief problem in the care of the deciduous teeth, and the maintenance of perfect comfort in mastication till the permanent teeth are erupted, relates to the proper management of the deciduous molars when they have become decayed. The deciduous incisors are usually lost and replaced by permanent ones at least five or six years before the molars are shed, and those years are sometimes very trying both to patient and practitioner. It is becoming quite generally recognized in the profession, and in fact to a large extent among our patients, that it is a matter of great importance to keep the deciduous teeth well cared for till they are lost by natural processes.

Aside from the resultant pain induced by exposed pulps and acute alveolar abscesses, and the constant absorption of pus into the system from chronic abscesses, there is another train of evils following neglect of the deciduous molars which is even more far reaching in its effect than either of these. This relates to the habits of mastication formed during youth as influenced by the condition of the deciduous teeth. If a deciduous molar is allowed to decay to any depth, it naturally becomes sensitive to hte impact of food. It takes the little patient but an instant to recognize the fact that to bite on that tooth results in discomfort, and a few twinges following the attempt to masticate on this particular side of the mouth are sufficient to relegate all of the food to the opposite side. Unilateral mastication does not subserve the full functional activity which nature intended in the proper management of the food by the teeth in its preparation for the stomach. More particularly is this the case in the mouths of children, where the total area of mastication is not very great, and where any reduction of this area proves serious. But worse than this, if a molra on each side of the mouth becomes involved—as is frequently the case—there is a very material crippling of the process of mastication, and the patient, not having arrived at years of accountability, has only one resource—the avoidance of mastication. Mastication hurts, and the only way to keep from being hurt is not to masticate. The result of all this is that the child begins bolting the food without chewing it, and the ultimate effect is to form a habit which may last through life.

If we study carefully the methods of mastication among adults, we shall find that there is a great variation in the thoroughness with which this function is performed—even among those who have relatively the same masticating equipment. If two individuals having equally good teeth show a striking variation in mastication, it must be due wholly to habit, and it is only reasonable to suppose that this habit is formed for the most part during youth. It will thus be seen that even apart from any consideration of the patient's present comfort and health, the maintenance of the deciduous molars n a condition of normal serviceability is a matter of very great importance.

In considering the care of these teeth it may be well to study separately the different classes of cavities and the different conditions manifested during the progress of the disease. In doing this no attempt shall be made to take up a certain phase of the subject, which, though having a very intimate bearing on the management of children's teeth, is of a character unsuited for presentation before a body of experienced practitioners. This relates to the control of the patient, the study of temperament as it affects the handling of these cases, and the general comity between patient and practitioner which enables the latter to carry out his work to the best advantage. All of this is something for each individual to study for himself, and it simply resolves itself into one of the chief requisites for the successful practice of dentistry in any of its departments, viz.: an intimate knowledge of human nature.

Taking up the technical procedures of the work then, the first class of cavities to be considered will be small occlusal cavities.

The control of this class of decay is usually not a very serious matter, and yet it is important that these cavities be given the closest attention. The direct impact of food on the occlusal surface renders a cavity in this region especially susceptible to discomfort during mastication, and it should therefore receive adequate protection. Probably the most serviceable filling material for these cavities is amalgam, though there are some cases in which, on account of the sensitiveness of the cavity or nervousness of the patient, no metal filling can be successfully used. It is, of course, not necessary to form cavities with the same degree of thoroughness demanded of operations on the permanent teeth, and yet if we use amalgam we must at least remove the decay. To do this in some cases is difficult, particularly when the cavity has long been exposed ot the fluids of the mouth and is exceedingly sensitive. To place oxyphosphate of zinc over a mass of decay and leave it for any time is hazardous, while to insret it temporarily with the idea of substituting it with something else n a few weeks after the sens t veness is relieved involves the difficulty of removing it. The best plan of procedure in these sensitive cases is to flood the cavity well with one of the essential oils, and then pack it with gutta percha for a week or ten days. Usually at the end of this time the gutta percha may be readily removed and the decay taken out with little discomfort. Pink base-plate gutta percha is best for this purpose, but in case the tooth is so sensitive that it cannot tolerate the heat and pressure necessary to manipulate the base-plate, some of the softer temporary stoppings requiring very little heat to soften them may be used.

The only preparation the cavity needs for filling aside from the removal of the decay is to break down thin enamel walls which overhang the cavity and make reasonably strong margins. In doing this hand instruments are usually preferable to the engine, though in certain cases a bur may be used to advantage if the patient is not too much frightened by it. In breaking down thin enamel around an occlusal cavity great care should be exercised not to allow the chisel to impinge against the interior of the cavity. This is usually very painful and unnerves the child. The best means of doing this work is with a very short bladed hatchet or hoe excavator, the shank of which will impinge against the outer surface of the tooth as each piece is cleaved away and thus prevent the blade from coming against the sensitive tissues of the cavity.

The fact that the crowns of these teeth are very short renders it impossible to admit of much decay on the proximal surface without involving the occlusal surface, so we are seldom called upon to prepare simple proximal cavities. The operator is fortunate if he catches the case shortly after the occlusal wall is broken in and before the pulp is involved. If only one tooth is decayed the problem of filling is simplified, but when the contiguous surfaces of both molars are gone it becomes vastly complicated. In case there is only one cavity to deal with and the pulp not involved the most serviceable material to use is amalgam, but where the pulp is too close to admit of metal filling, the choice must be between gutta percha and cement. These materials are temporary in their nature, but there is this advantage in gutta percha over cement; that when it fails it is not deceptive in its method of failure as is sometimes cement. Gutta percha wears away on the exposed surface more rapidly than cement, but it seldom admits of a pronounced excavation under the filling. With cement we may find a filling badly undermined at the gingival margin even to the point of pulp exposure when from the occlusal aspect the filling appears in good condition. But on the other hand there are some of these cavities of such a form that gutta percha will not remain in them, and so sensitive that they cannot be formed to retain it. In these cases cement may be made to do service by virtue of its adhesive properties, and the difficulty at least tided over temporarily till the tooth is in a condition to accept more permanent work.

In cases where both molars are involved and the cavities face each other, a very serious complication at once manifests itself. If we restore the original contour of the tooth with the limited anchorage which we are usually able to get in these sensitive cases, we are liable to have the fillings loosened by the tipping stress exerted in mastication; and if we do not contour, we at once subject the patient to all the annoyance and pain of having food wedged between the teeth into the interproximal space. This matter of the wedging of food between the deciduous molars is really responsible for much of the complaint made by children during mastication, and to overcome this difficulty is one of the chief problems presented to us. In some very persistent cases it seems impossible of accomplishment, short of bridging across from one tooth to the other in a solid mass of filling. This method has serious objections and should be resorted to only in those desperate cases where nothing else promises relief. When it is attempted, the filling should be preceded by a small flat metal guard laid across the interproximal space over the gum, and resting one end on the gingival wall of one cavity and the other on the gingival wall of the other. Over this the filling may be built with the utmost assurance that the gum will be perfectly protected against injury and the teeth made comfortable. These metal guards may be cut from hin clasp metal or german silver, and before being placed in position a small bit of gutta percha may be stuck to each end and warmed so that when the guard is pressed against the gingival wall of the cavity the gutta percha will seal the space between the guard and the cavity.

As to the kind of filling material to be used in this bridging process we are practically confined to two—gutta percha and amalgam.

Cement is almost worthess for this purpose. It is so rigid that it will not in the least accommodate itself to the indivdiual movement of the teeth during mastication as will gutta percha, and it is not sufficiently trong to hold firm when pressure is brought to bear upon one tooth and not on the other. The consequence is that in a very short time we find it loosened from one of the cavities and frequently from both. The same difficulty arises, though in a less degree, with amalgam. But amalgam is much stronger than cement, and if we can get deep anchorage for it in both cavities it will sometimes remain secure for an appreciable time. Gutta percha s the best material for this purpose with the one limitation that it wears away quite rapidly and calls for periodical renewal. But meanwhile the teeth are kept comfortable for mastication, which is an important consideration, and it would seem preferable in desperate cases to see the patient every two or three months and renew the gutta percha rather than resort to methods which so often prove disastrous.

In cases where the cavities are filled without bridging, it is always best if amalgam is used to fill one cavity at one sitting and the other at a subsequent one. This admits of giving the proper contour to the first filling and so rounding out the contact point and polishing it after it has become hard that the second filling may be conveniently built against it and trimmed to the best advantage. If this is attempted with both fillings at once while the amalgam is soft, it usually results in imperfect contact or in an appreciable space between tho fillings.

When a pulp becomes exposed in one of the deciduous molars the problem arises as to how it shall best be treated. Usually these pulps are not very tenacious of life and so are not susceptible to treatment for their preservation. And yet it would seem undesirable, in view of all the conditions surrounding the case, to make an application of anything so powerful as arsenic to destroy a pulp in a deciduous tooth. The distance from the point of exposure to the apical foramen is not very great, and the vascularity in these young tissues is more pronounced than in adult life, so there is always the possibility of the effects of the arsenic being carried beyond the tooth into the apical space. This is especially true if absorption of the end of the root has begun preparatory to the admission of the permanent tooth. Added to this is the difficulty of securing perfect sealing of the agent in these young mouths where the control of the patient is usually not so certain as with adults, and where the area between the point of exposure and the gingival margin of the cavity is exceedingly limited. If an exposure occurred in an occlusal cavity and the pulp were very troublesome and persistent of life, there might be some justification for applying a minute quantity of arsenic, but these conditions so seldom arise that it may be stated as a safe rule never to apply arsenic to a deciduous tooth. The seriousness of the effects of arsenic when carried beyond the apex in children's teeth may easily become very great, and the necessities of the case do not ordinarily call for so evident a risk. As has been stated, these pulps do not die hard, and if given a reasonable chance they will ordinarily disintegrate of their own accord. The problem is merely to keep them comfortable during this process, and this can usually be done in the following manner:

When an exposed and inflamed pulp is brought for treatment, the first procedure is to syringe out the cavity with warm water and remove all of the debris and decalcified dentine with an excavator. This may readily be done in these cases, with almost no pain, if the operator is careful not to touch the pulp. After the cavity is cleaned as perfectly as possible, it is usually best to slightly puncture the pulp with a fine explorer ot induce a free flow of blood. This may cause momentary pain but it is only momentary and subsequent relief is thereby assured. When the pulp begins to bleed, warm water should be gently syringed into the cavity till the bleeding ceases, by which time the tooth will usually be comfortable.

Make a paste of the oil of cloves with the powder supplied with our oxyphosphate of zinc, and gentlyp at it over the exposure and seal the cavity carefully with gutta percha. A pulp treated in this way will remain comfortable till it dies, and the first indication of death is a slight soreness in the tooth on pressure. Instruction should therefore be given to bring the little patient to the office on the first symptom of soreness, when the gutta percha may be removed and the canals treated in the usual way. Of course, there is occasionally the possibility of the case being neglected, as other cases often are, till an abscess starts, but this is a contingency for which the dentist is not responsible, and usually if an abscess does begin it can readily be relieved by opening the cavty to give it vent, syringing it out well with warm water and applying an antiseptic.

The treatment of pulpless deciduous teeth is little different in plan from that of permanent teeth with the exception of two features of the case which need mention. A deciduous tooth, the canals of which have been exposed for any length of time to the fluids of the mouth seems to become more extensively saturated with the products of decomposition than a permanent tooth under similar conditions. There is nothing viler ever tolerated in the human mouth than a long exposed putrescent deciduous molar. It taints the breath of the little patient, and the first stirring up of the contents of such a tooth usually permeates the air of the operating room for some distance. In addition to this there is ordinarily greater difficulty in securing a perfect mechanical and medicinal cleansing of the cavity and canals in a child than in an adult on account of the limitations under which the operator must work. It is not often possible to apply the rubber dam, and the problem of excluding fluids of the mouth during the treatment of the case is difficult. In view of these conditions it is frequently necessary to extend the treatment over a greater length of time, and to change the medicaments oftener than usual in order to overcome the putrescence and render the tooth fit to receive the filling.

In cases of abscess with fistulous opening, if the fistula does not heal after the canals seem in good condition and medicine has been forced through the fistula once or twice, it is sometimes a very effective method to flood the canals with a solution of gutta percha in eucalyptol and pump this through the fistula, following it immediately with a solid gutta percha root filling. The reason for not doing this in the first instance is because these putrescent cases should be kept under the nfluence of an antiseptic for at least a week before attempting to fill the roots. If the canals have been made aseptic, the fistula will usually heal, following the passage of the eucalyptol solution.

In discussing pulpless deciduous teeth there is one brief reference which would seem desirable before closing the subject. The question has sometimes arisen as to the effect of the loss of the pulp on the process of root absorption preparatory to the eruption of the permanent teeth, and the statement has been made that roots would not be absorbed if the pulps were destroyed. This is a manifest error, as has been amply demonstrated by the exhibition of deciduous crowns which have toppled out with the roots completely absorbed and the gutta percha root fillings still clinging to the crowns. But it is a different matter if the teeth are allowed to remain in the mouth badly abscessed. With the ends of the roots constantly bathed in pus the normal process of absorption cannot be expected to go on, and this is only one additional argument why diseased deciduous molars should be restored to a condition of health.

